# Supplementary material for: Multiscale compression-induced restructuring of stacked lipid bilayers: From buckling delamination to molecular packing
Source: PLoS One. 2022 Dec 9;17(12):e0275079. doi: 10.1371/journal.pone.0275079 (PMC9733850; doi:10.1371/journal.pone.0275079)
Supplement: S1 File — (PDF) [file pone.0275079.s001.pdf]

S1 Supporting Information. Platform for inducing compression

For the platform to induce compression on the lipid films, we utilized a glass slide as a platform and tape to hold the PDMS (Fig A). The dimensions of the platform are 38 mm x 13 mm. The area of PDMS located at the center of the platform is 13 mm x 13 mm. As sliding the top glass slide attached on the bottom glass slide, PDMS was able to be stretched or compressed (Fig B). The process for inducing compression on lipids is that, first, we applied strain to the PDMS and held it in place using tape, second, we deposited the lipid film either by spin-coating or drop-casting, finally, we applied the compressive strain by releasing the tape holding the PDMS (Fig C). We used a new platform for each sample.

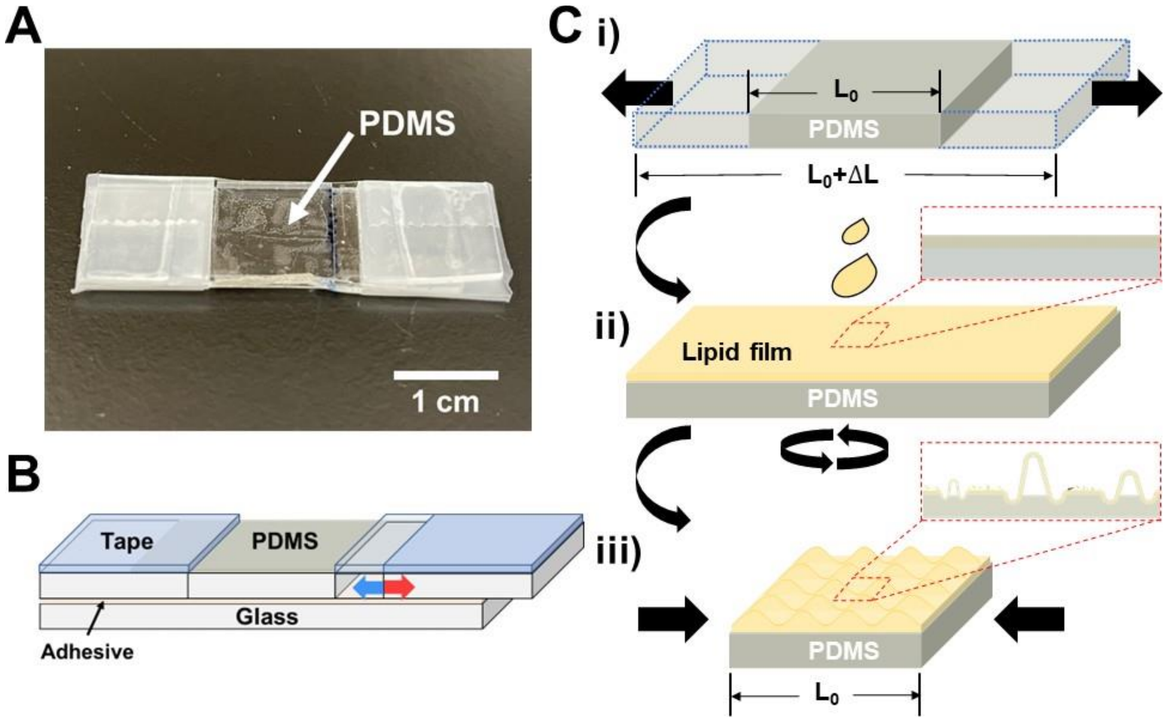

Figure. **Platform and process for inducing compression.** A) The optical image of the platform. B) The corresponding schematic of the platform. The red and blue arrows indicate the stretching and compression of PDMS, respectively. C) The schematic of the process for inducing compression. i) The PDMS is stretched to a specific percentage of its original length. ii) The lipid film is deposited on the PDMS. iii) The applied strain is released leading to compression of the PDMS and hence the lipid film.
