# Supplementary material for: Multiscale compression-induced restructuring of stacked lipid bilayers: From buckling delamination to molecular packing
Source: PLoS One. 2022 Dec 9;17(12):e0275079. doi: 10.1371/journal.pone.0275079 (PMC9733850; doi:10.1371/journal.pone.0275079)
Supplement: S2 File — (PDF) [file pone.0275079.s002.pdf]

S2 Supporting Information. X-ray Scattering Data Analysis

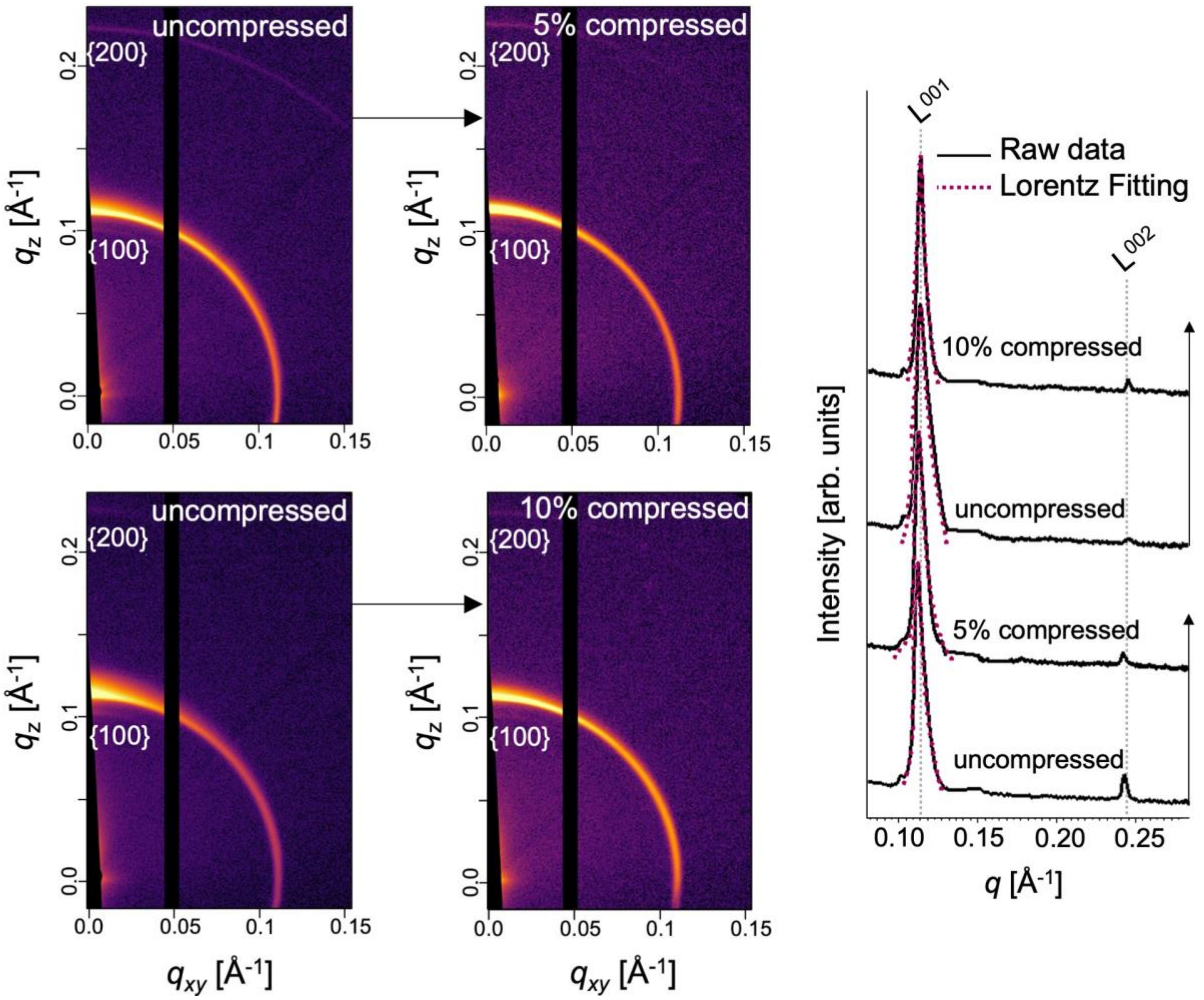

Figure A. Structure of DPPC films as a function of compression. 2D GISAXS diffraction patterns of DPPC supported lipid multilayered (SLM) films before and after compression by 5% and 10%. The brackets denote family of planes *hkl* (left). Corresponding linear integration of diffraction patterns (right).

We calculated structural parameters of lipid films from GISWAXS data (Table A). We compared DPPC against DOPC films deposited on rigid (SiO_2) or flexible PDMS substrates. From GISAXS, we estimated inter-bilayer distance d, FWHM and correlation length, ξ related to the domain size of the lipid layers. From GIWAXS, we obtained distance between lipid tails a, FWHM, and correlation length, ξ^{φ} related to the degree of order at the lipid tail length scale.

Lipid	Substrate		SAXS peaks d [Å]	SAXS peaks FWHM×10^3 [$Å^{-1}$]	SAXS peaks ξ [Å]	WAXS peaks a [Å]	WAXS peaks FWHM×10^3 [$Å^{-1}$]	WAXS peaks ξ^{φ} [Å]
DPPC - L_β	SiO_2		55.7	3.08	2040	4.12	134	50
	PDMS	PDMS-uncompressed	56.2	2.44	2579	4.12	61	103
		PDMS-5% compressed	56.0	2.28	2776	4.11	76	83
		PDMS-uncompressed	56.4	2.24	2811	4.15	61	103
		PDMS-10% compressed	56.8	2.17	2896	4.12	66	95
DOPC - L_α	SiO_2		52.6	2.28	2756	4.44	523	12
	PDMS	PDMS-uncompressed	51.9	3.07	2045	4.51	562	11
		PDMS-5% compressed	51.9	2.62	2398	4.47	841	8
		PDMS-uncompressed	52.1	2.54	2479	4.40	528	12
		PDMS-10% compressed	51.9	2.08	3028	4.32	633	10

Table A. Lipid film parameters obtained from GISWAXS data

We scanned the bare PDMS substrate with X-rays to exclude PDMS peaks from our SLM film analysis (Figure B).

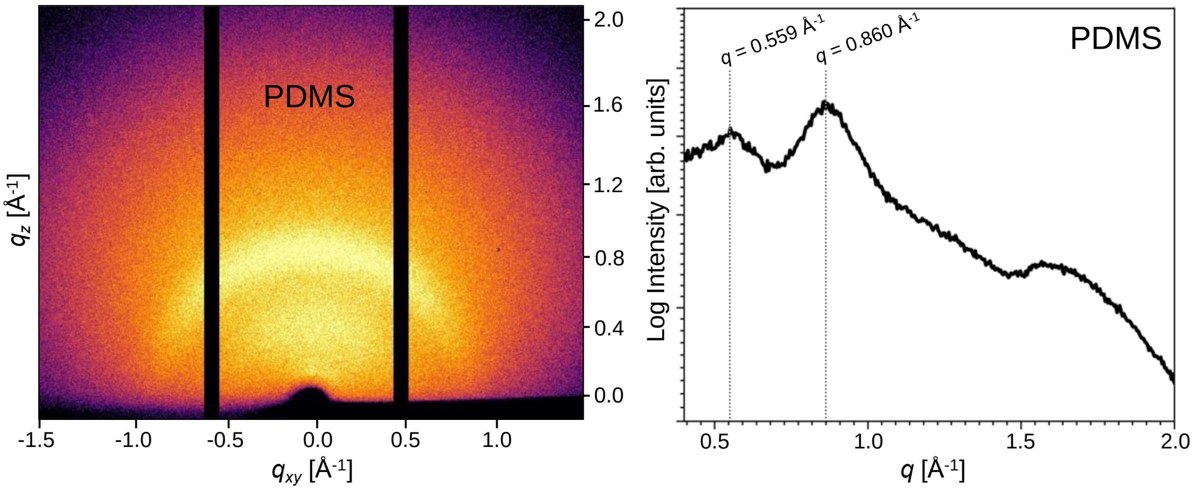

Figure B. X-ray structure of PDMS. 2D GIWAXS image of PDMS (left) and corresponding linearly integrated GIWAXS data (right).

Reconfiguration of a fluid L_α phase SLM film

Contrary to DPPC, DOPC is in a disordered state at room temperature (T > Tm). Hence, we used DOPC multilayered films to compare the effect of compression in a fluid phase. We evaluated the effect of compression on DOPC SLM films at ambient temperature using grazing incidence small and wide-angle X-ray scattering (GISWAXS). From the integrated X-ray data, we fitted the peaks to a Lorentzian function and extracted the peak position and full width half max (FWHM).

GISAXS data reveal that DOPC SLM films exhibits a rhombohedral phase (data not shown here) with an average lattice parameter *d* = 5.2 nm which remained unchanged upon compression. Regarding the correlation length for lipid bilayer orientation, DPPC has higher correlation length (ξ = 103 ˚A) compared to DOPC (ξ = 11 Å). In contrast to gel phases, Lα such as DOPC display a broad peak at $q \approx 1.5$˚$Å^{-1}$ characteristic of the liquid nature of the DOPC films. The average separation between the hydrocarbon chains is larger (*a* = 4.4 Å) than DPPC's (*a* = 4.1 Å) since DOPC carries two double bonds making the molecules adopt a Gauche conformation hence taking up larger lateral space.

Similar to DPPC, the order parameters for DOPC change upon compression. The domain size of DOPC multilayers increases from ξ = 248 nm to 303 nm with 10% compression. We interpret the difference in ξ as an increase of domain size of 55 nm i.e., approximate 11 more lipid bilayers aligned at the same orientation. Moreover, in the same way as DPPC, for DOPC compression decreases ξ^{φ} from 11 to 8 ˚A upon 5% compression, indicating a higher degree of disorder in the L_α phase, where the lipid chain orientation becomes more variable. In conclusion, compressive strain has similar quantitative effects on lipid multilayered films in both the fluid L_α and gel L_β phases.

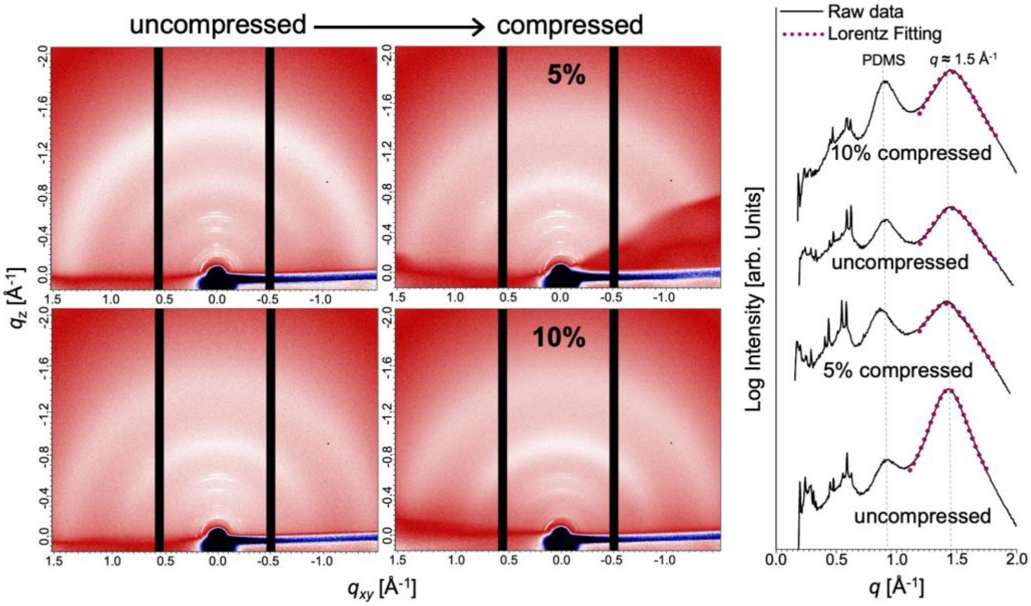

Figure C. Structure of DOPC SLM films as a function of compression. 2D GIWAXS diffraction patterns (left) of DOPC films before and after compression by 5% and 10%. Corresponding linear integration of diffraction patterns (right) (integrated intensity vs scattering vector *q*).
