# Supplementary material for: Multiscale compression-induced restructuring of stacked lipid bilayers: From buckling delamination to molecular packing
Source: PLoS One. 2022 Dec 9;17(12):e0275079. doi: 10.1371/journal.pone.0275079 (PMC9733850; doi:10.1371/journal.pone.0275079)
Supplement: S3 File — (PDF) [file pone.0275079.s003.pdf]

S3 Supporting Information. Derivation of adhesion energy between supported lipid films and PDMS

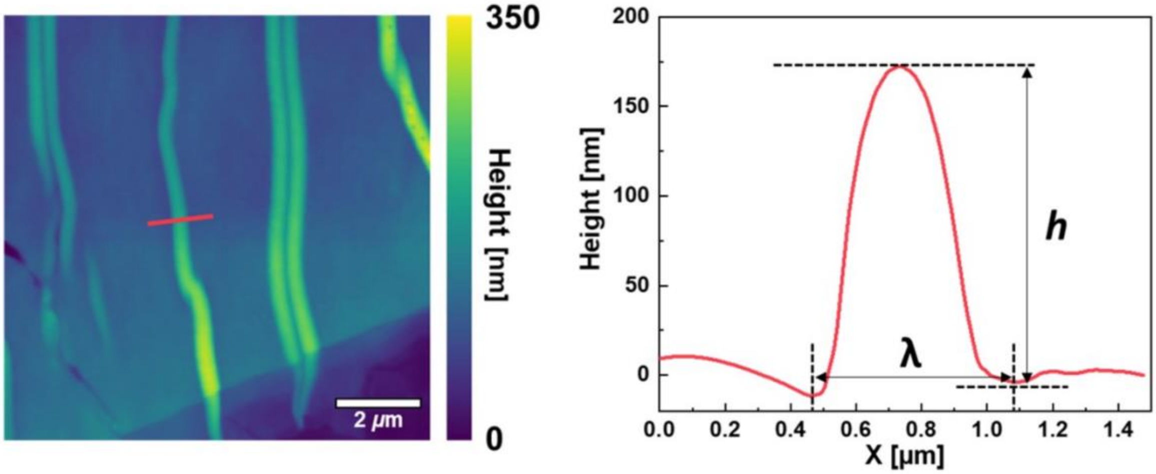

Figure. Topography of delamination buckles. AFM topography image of delamination buckles (left) and profile of delamination buckles corresponding with the red line in the left AFM image.

To calculate the adhesion energy between lipid films and the PDMS substrate, we introduce the reported energetic model for thin film on soft substrates[1]. The total energy per unit width (E_{total}) of the isotropic materials system is composed of elastic energy stored in the substrate ($E_{substrate}$), bending energy ($E_{bending}$) and stretching energy ($E_{stretching}$) in the film, and adhesion energy between the film and the substrate ($E_{adhesion}$). Hence, E_{total} is estimated as:

$$E_{total} = E_{substrate} + E_{bending} + E_{stretching} + E_{adhesion} \tag{S1}$$

In this calculation, we follow the literature reported assumptions[1]:

1. The geometry of delamination buckle (z) with width (λ), and height (h), is characterized by a sinusoidal profile (Figure):

$$z = \frac{h}{2}[1 + \cos(\frac{2\pi x}{\lambda})]$$

2. The lipid film and PDMS substrate are both incompressible (Poisson's ratio ν = 0.5)
3. The lipid film is inextensible, so the stretching energy in the film is negligible (i.e., $E_{stretching} \approx 0$)

---

[1] Zhang, Q. and Yin, J., 2018. Spontaneous buckling-driven periodic delamination of thin films on soft substrates under large compression. *J. Mech. Phys. Solids.* 118, pp.40-57.

4. $E_{bending}$ is much bigger than $E_{substrate}$ and shear strain energy in the film. By applying these assumptions, E_{total} is simplified as:

$$E_{total} = E_{bending} + E_{adhesion} \tag{S2}$$

$$= \frac{\pi^4 E_f t^3 h^2}{12\lambda^3} + \frac{\Gamma\lambda}{1-\epsilon} = 0$$

The geometry of profile is determined by minimizing the total energy,

$$\frac{\partial E_{total}}{\partial \lambda} = -\frac{\pi^4 E_f t^3 h^2}{4\lambda^4} + \frac{\Gamma}{1-\epsilon} = 0 \tag{S3}$$

After cancelling *h* in equation (S3) using the geometric relationship in equation S4,

$$\frac{\lambda}{t^{1.5}} = \sqrt{\frac{\pi^2 \bar{E}_f \epsilon}{\Gamma}} \tag{S4}$$

the relationship among adhesion energy, geometric parameters, and plane Young's modulus is obtained as:

$$\frac{\lambda}{t^{1.5}} = \sqrt{\frac{\pi^2 \bar{E}_f \epsilon}{\Gamma}}$$
