# Supplementary material for: Multiscale compression-induced restructuring of stacked lipid bilayers: From buckling delamination to molecular packing
Source: PLoS One. 2022 Dec 9;17(12):e0275079. doi: 10.1371/journal.pone.0275079 (PMC9733850; doi:10.1371/journal.pone.0275079)
Supplement: S4 File — (PDF) [file pone.0275079.s004.pdf]

S4 Supporting Information. Time-dependent relaxation of supported lipid films

We quantified the time dependence relaxation of the surface patterns of DPPC supported lipid multilayered (SLM) films. From these results, we found that inelastic relaxation of DPPC SLM films occurs over the time scales of hours to days after compression. To ensure measurements across different techniques may be correlated, we perform all measurements discussed here within 1 hour of initial compression. AFM topography shows the lipid covered regions have higher topography than the PDMS uncovered regions (Figure A). After 24 hours, lipid materials filled up the PDMS exposed regions, and the wrinkles and smaller delamination buckling disappeared, and large delamination buckling became wider and shorter. We confirmed with confocal laser scanning microscopy (CLSM) that the amount of lipid material distribution does not change significantly over time (Figure B). Even though the lipid films exhibit mechanical properties that match with the continuum elastic model right after compression, the relaxation of the lipid film over time exhibits some inelastic behavior. This inelastic behavior is consistent with the observation of molecular lipid tail disorder after compression.

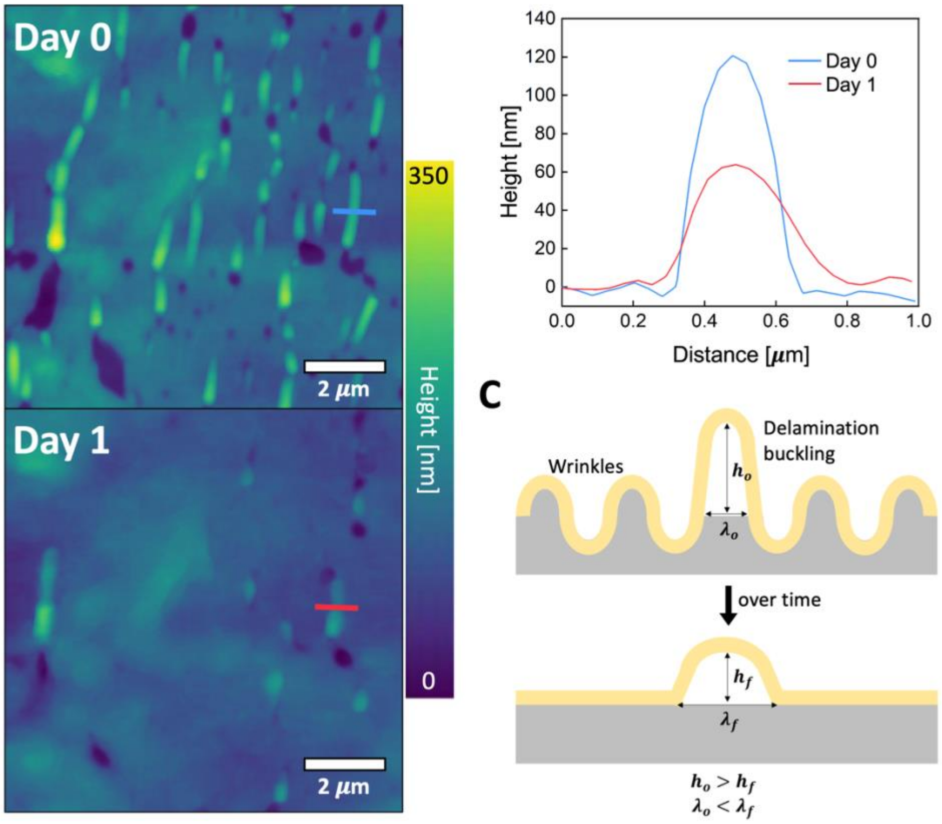

Figure A. Relaxation of lipid film morphology over time AFM topography images (left) right after compression (Day 0) and 24 h after (Day 1) with cross-section height profiles of lines indicated in the AFM image, and schematic representation of relaxation of the lipid film over time (right).

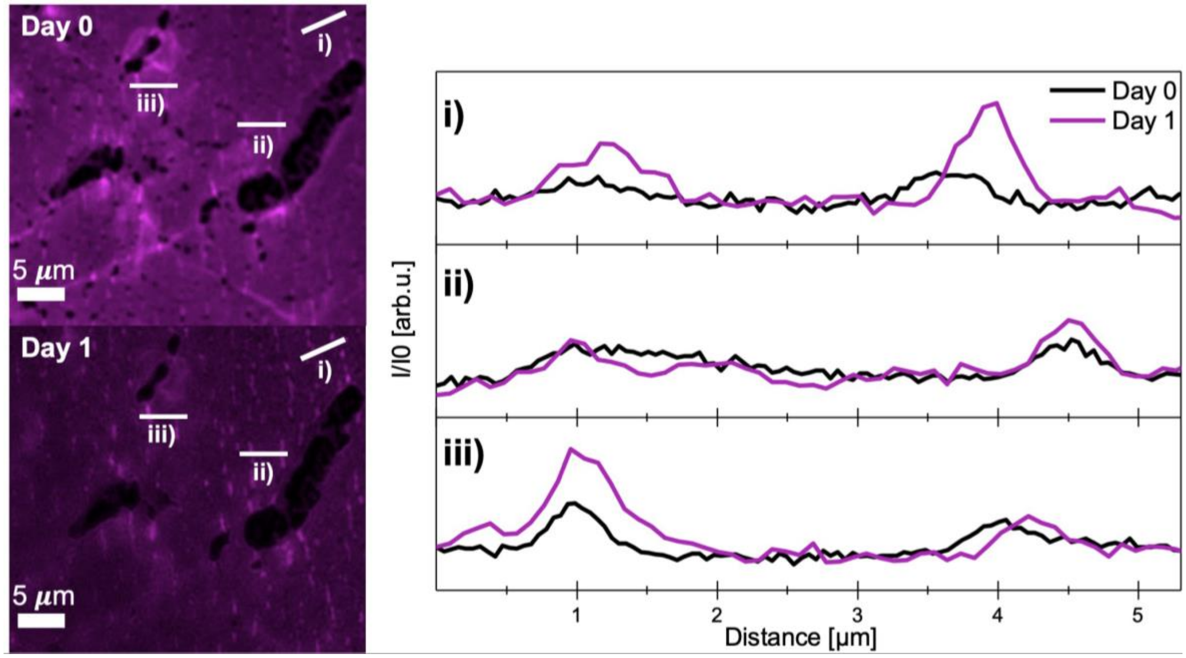

Figure B. CLSM morphology change over time. To visualize the morphology of lipid film after compression, we added Rhod-PE fluorescent probe to the lipid film to distinguish between lipid material and the PDMS substrate. 2D CLSM images (Scale bar: 5 μm) right after (Day 0) and 24 h after (Day 1) compression by 20% (left). The magenta regions indicate enriched domains of the DPPC film, while the dark areas correspond to PDMS exposed regions where there is no lipid coverage. The bright vertical lines in the images correspond to delamination buckling regions displaying higher fluorescence intensity than flat regions. Normalized fluorescence intensity profiles obtained from different areas of CLSM images show that the fluorescence intensity of delamination buckling regions is almost identical after 24 hours which suggests that the amount of lipid distribution remains constant over time (right).
