# Supplementary material for: Multiscale compression-induced restructuring of stacked lipid bilayers: From buckling delamination to molecular packing
Source: PLoS One. 2022 Dec 9;17(12):e0275079. doi: 10.1371/journal.pone.0275079 (PMC9733850; doi:10.1371/journal.pone.0275079)
Supplement: S5 File — (PDF) [file pone.0275079.s005.pdf]

S5 Supporting Information. Laurdan data analysis

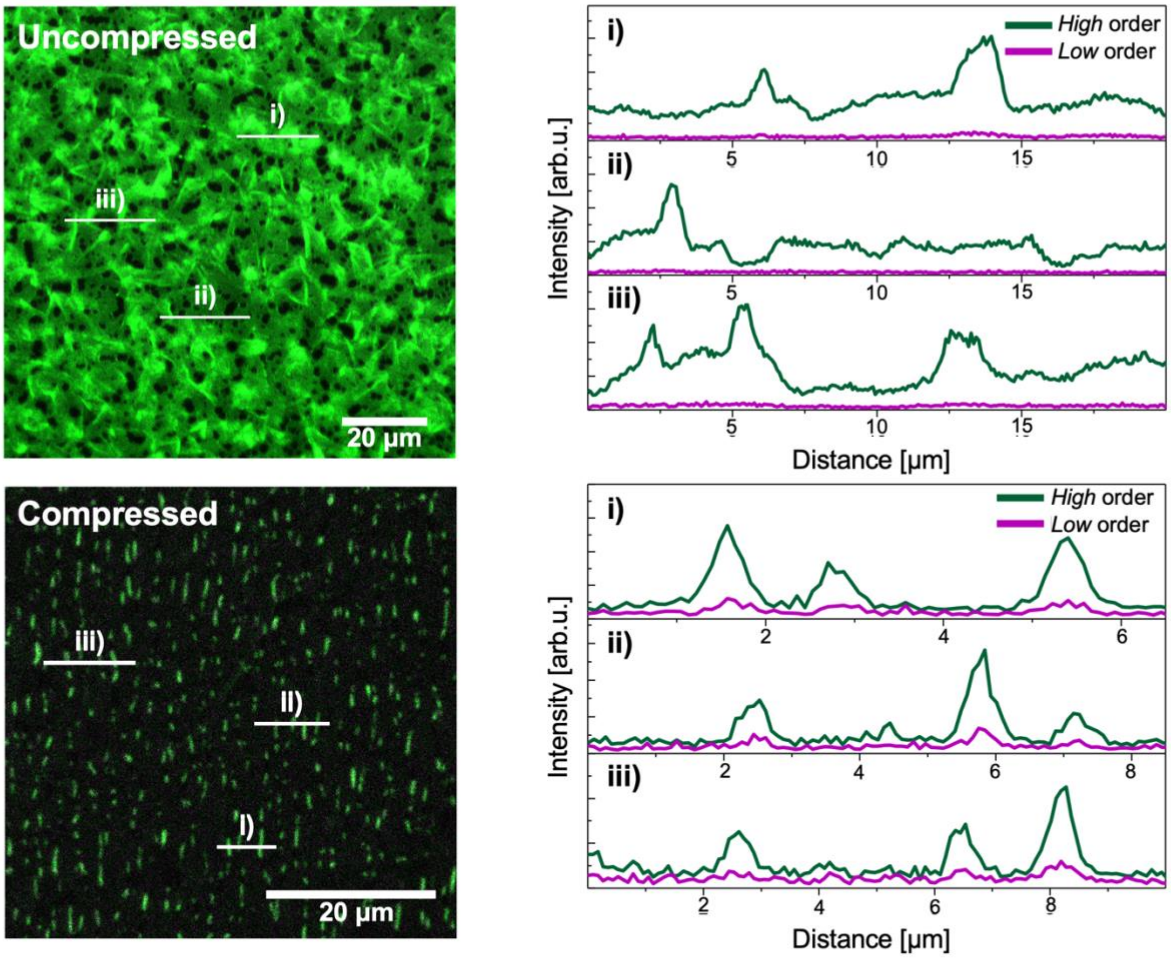

Figure A. Optical analysis of hydrocarbon chain order of DPPC supported lipid multilayers (SLM) films upon compression. Confocal laser scanning microscopy (CLSM) images of DPPC-Laurdan SLM films adsorbed onto PDMS before and after compression by 20% (left). 1D intensity profiles along the white lines of each Laurdan dye channel (*high* ordered hydrocarbon chains fluorescence in green and *low* ordered hydrocarbon chains fluorescence in magenta (right).

Laurdan emission wave interference

To rule out thin film wave interference of magenta (*high* order) and green (*low* order) emission channels due to buckle delamination (Figures A), we calculated constructive and destructive interference conditions for both emission wavelengths. The max neutralizing effect, meaning complete annulment, happens when waves are 180°out of phase.

$$\Delta\phi = 180^\circ = \pi$$

$$\Delta d = \frac{\Delta\phi\lambda}{2\pi}$$

$$\Delta d = \frac{\pi\lambda}{2\pi} = \frac{\lambda}{2}$$

Spacing for *high* order emission wavelength:

$$\Delta d = \frac{450nm}{2} = 225nm$$

Spacing for *low* order emission wavelength:

$$\Delta d = \frac{500nm}{2} = 250nm$$

Since the spacing between layers in the SLM film is:

$$\Delta d \approx 100nm$$

emission waves are not completely out of phase, meaning they are not completely destructive.

We performed Laurdan spectral analysis in ambient environment for pure DPPC and DOPC lipids (Fig. B), which are representative of *high* and *low* order membranes at room temperature, respectively. Samples were excited with Xenon lamp excitation at λ_{ex}=350 nm (bandpass: 20 nm) and the emission spectra was collected in n=3 measurements. DPPC at low temperature (25 °C) is assembled in a bilayer where hydrocarbon chains have a high degree of order yielding a Laurdan emission spectra with a maximum at ca. 443 nm. DOPC at 25 °C (above its transition temperature) comprises hydrocarbon tails with more conformational freedom yielding a peak with a maximum at ca 480 nm.

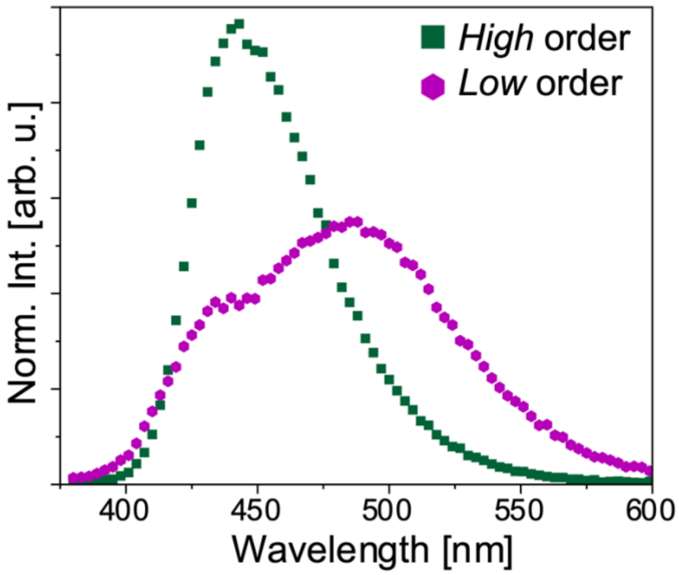

Figure B. Emission spectra of 1 mol% Laurdan in 50 mM DPPC and DOPC samples at 25 °C. Both samples were excited at 350 nm (20 nm bandpass) with Xenon lamp and corresponding spectra was collected using Synergy Neo 2 microplate reader (Biotek). DPPC has a *high* order phase Laurdan emission spectra with a peak at ca. 443 nm (colored in green). DOPC has a *low* order phase with a peak at ca. 480 nm (colored in magenta).
