# Supplementary material for: Multiscale compression-induced restructuring of stacked lipid bilayers: From buckling delamination to molecular packing
Source: PLoS One. 2022 Dec 9;17(12):e0275079. doi: 10.1371/journal.pone.0275079 (PMC9733850; doi:10.1371/journal.pone.0275079)
Supplement: S6 File — (PDF) [file pone.0275079.s006.pdf]

## S6 Supporting Information. Simulations

Figure A shows the formation of undulations in a Lα phase DOPC multilayered membrane. The lipids in the snapshots are color coded to represent their instantaneous tilt angles with respect to z-axis. Figure C shows the increase in the correlation of lipid tilts with strain, across layers in multilayered DPPC and DOPC membranes.

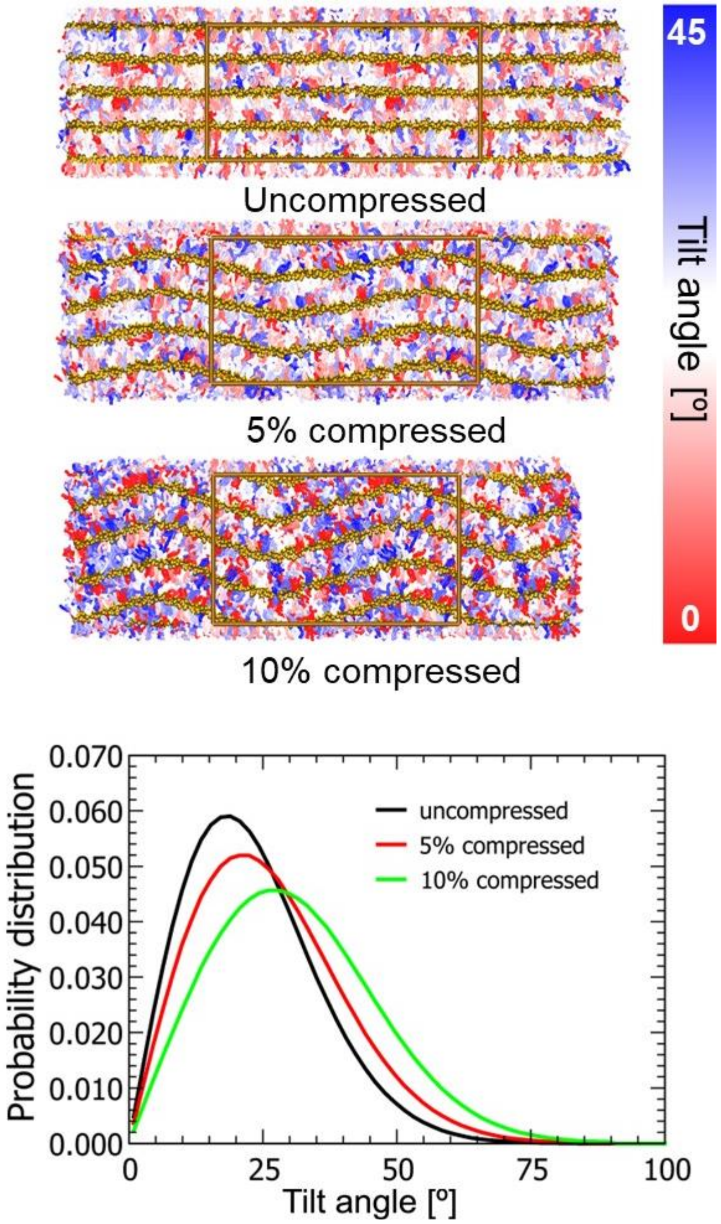


**Figure A.** Effect of compression on lipid tilt. Color coded representation of the simulated DOPC systems at three compressive strains (top). The color represents lipid orientation (tilt) angle with respect to z-axis. The box represents the simulation unit cell. The tilt angle distributions of the $L_\alpha$ phase DOPC lipids (bottom).

Order-parameter was computed for individual bonds of MARTINI DPPC and DOPC (Figure B) lipids for different strains. The reduction in order in lipid tail bonds (# 3-10) for both DPPC and DOPC is directly observed. Order-parameter was also computed using a lipid director approach to isolate the lipid orientation (tilt) and tail conformation parts of order-parameter. We observed that the change in tail conformational order with strain is negligible for both DPPC and DOPC lipid systems.

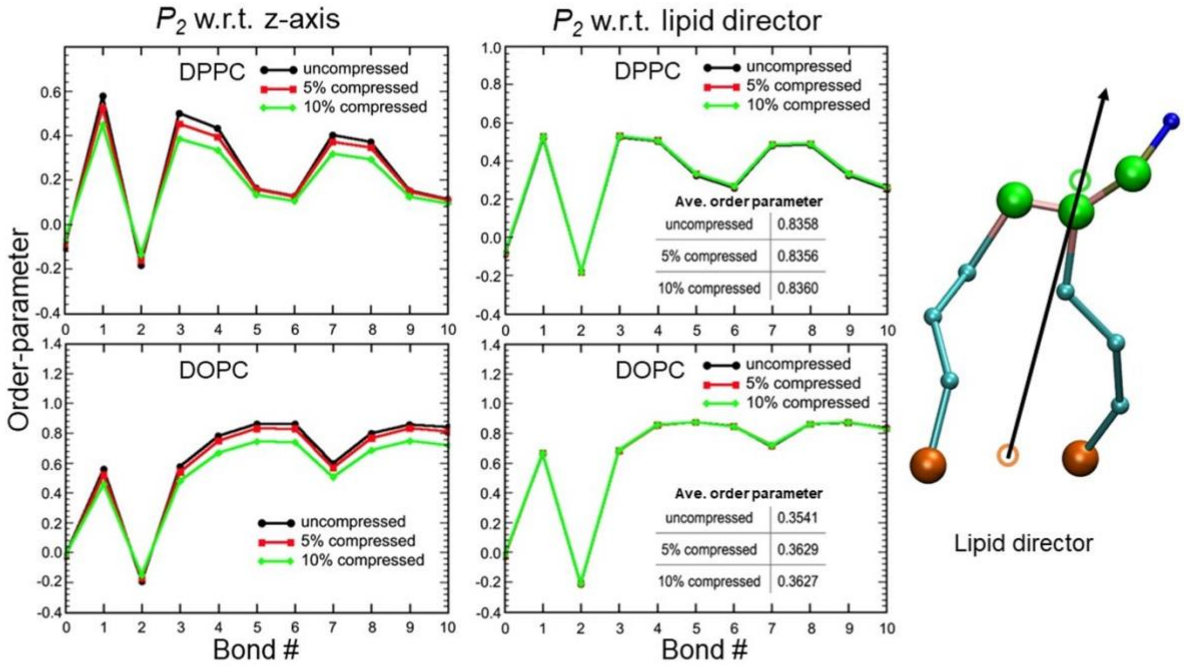


**Figure B.** Order-parameter ($P_2$) plots. $P_2$ computed using z-axis as reference normal for DPPC (top-left) and DOPC (bottom-left). $P_2$ computed using lipid director as reference normal for DPPC (top-center) and DOPC (bottom-center). Insets show the average order-parameter values. Representation (right) of lipid director (black arrow). Relevant head groups (PO4, GL1, GL2 in MARTINI representation) and tail groups (C4A, C4B in MARTINI representation) are depicted as green and orange spheres, respectively.

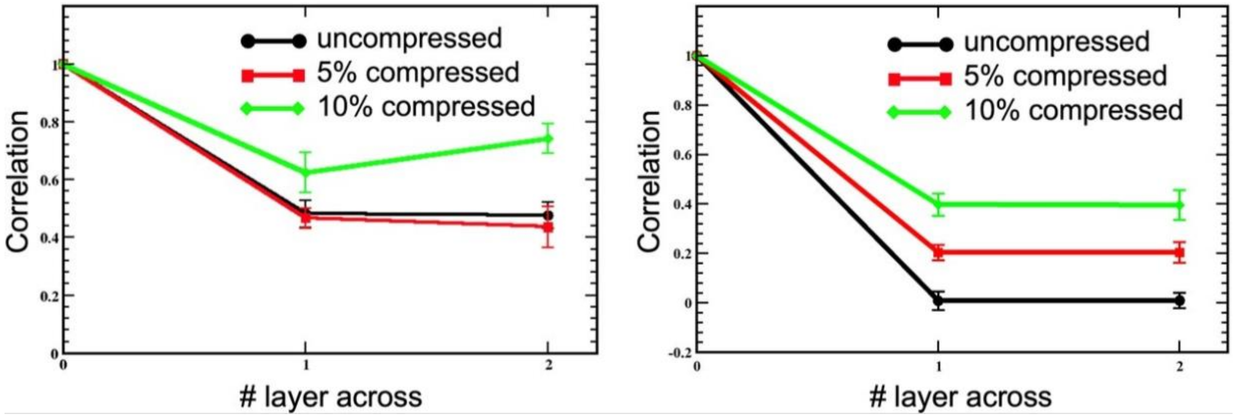


**Figure C.** Normalized inter-layer tilt correlation values for DPPC (left) and DOPC (right) multilayered membranes subjected to compressive strains.
