# Supplementary material for: Multiscale compression-induced restructuring of stacked lipid bilayers: From buckling delamination to molecular packing
Source: PLoS One. 2022 Dec 9;17(12):e0275079. doi: 10.1371/journal.pone.0275079 (PMC9733850; doi:10.1371/journal.pone.0275079)
Supplement: Attachment — Submitted filename: Response to Reviewers.pdf [file pone.0275079.s007.pdf]

We are thankful to the Editor for giving us the opportunity to revise our manuscript. The Reviewer recommended minor revisions and provided important points for improvement. We clarify previously confusing or incomplete statements and expanded on our results and discussion to provide a revised document that fully addresses all concerns/comments/and suggestions raised by the Reviewer. In the report below we answer (in boxed text) to these concerns (in bold text) point-by-point and provide the corresponding changes made to the manuscript and the supporting material. We highlighted the changes made to the original version in the file labeled 'Revised Manuscript with Track Changes'.

Reviewer #1: This is an interesting and timely piece of work. As the author's state. There aren't many studies on mechanics of multilayer membranes. Below are some minor comments:

We thank the Reviewer for acknowledging the importance and potential impact of our work. We are particularly thankful for their careful assessment and constructive criticism that guided us to produce an improved version of our initial manuscript.

1) Can the authors state the controlled release increments of the substrate strain on page 3.

We thank the Reviewer for identifying a lack of explanation here. We have expanded the line to quantify the percentages we used to pre-strain the PDMS substrates. Lines 83-85 in page 3 now read: "Finally, we released the substrate pre-strain by controlled increments of 5, 10 or 20% to induce compression on the lipid film."

2) Explain the color coding in Fig. 1.

We appreciate the Reviewer's attention to detail. We have added an additional component to Fig 1 to enlarge a section of the simulated lipid multilayers with the objective of providing clarification for the color coding. We have also expanded the caption of Fig 1.

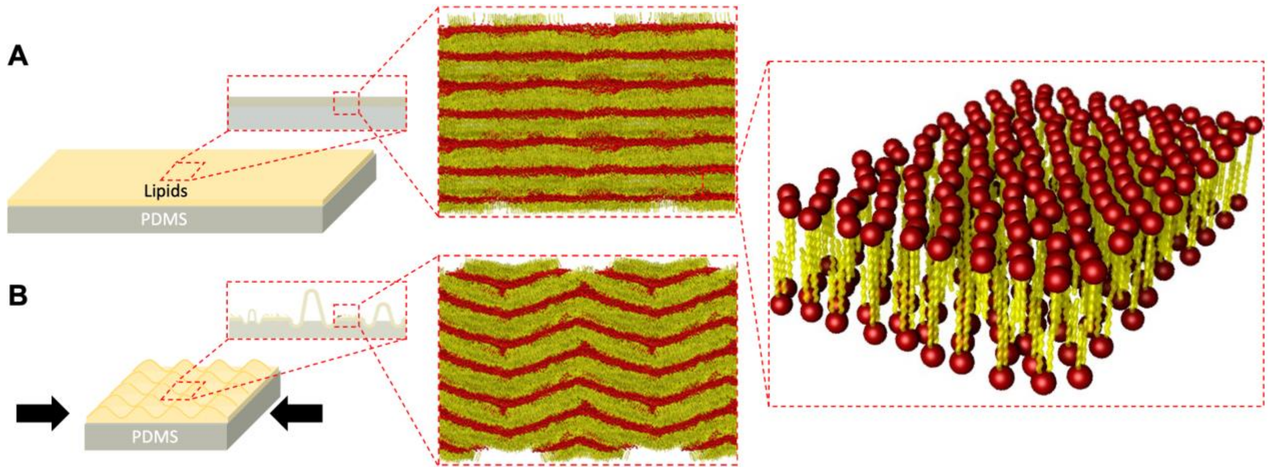

Fig 1. Substrate-mediated reconfiguration of lipid multilayered films. Schematics and simulations (discussed further in Fig 5) of the multiscale structure of multilayered lipid films on a deformable substrate before (A) and after (B) compression. The schematic in the right expands on a section of the lipid multilayered system to highlight a bilayer where headgroups are represented in red and tails in yellow.

3) Typos on line 139 and 155

CL: Department of Materials Science and Engineering • MSEB, 1304 West Green St, Urbana, IL 61801 • E: cecilial@illinois.edu • T: 217-300-1955 • W: http://www.matse.illinois.edu/faculty/Leal.html
AZ: Department of Mechanical Science and Engineering • 214 Seitz Materials Research Lab, 104 South Goodwin Ave, Urbana, IL 61801 • E: arendv@illinois.edu • T: (217) 244-2912 • W: https://vanderzandelab.mechse.illinois.edu

We thank the Reviewer for indicating the typos. Both have been corrected in the corresponding lines.

4) Revise line 198- is that the adhesion energy between the individual lipid membranes?

We appreciate the Reviewer for requesting clarification. The Reviewer is correct, the adhesion energy cited in line 198 correspond to adhesion between two lipid bilayers, and we have edited the sentence to clarify it, which now reads: “For comparison, the adhesion energy between two DPPC bilayers in water is 0.15 mJ/m^2.”

5) Laurdan experiments: I am not sure I see the high-intensity distinct domains on Fig. 4 A, C (line 242); There seems to be crosstalk between the two channels as they both show fluorescence at the same regions in the membrane. For this reason, I am not convinced that the increase in the magenta channel on 4B is due to increasing disorder. Can the authors use spectral imaging instead?

We are thankful that the Reviewer brings up this point. The crosstalk between the two detection channels was addressed by adding Laurdan spectroscopy data (*new* Figure B in S6 Supporting Information). There is a constrain in emission detection due to narrow spectral separation between *low* and *high* order domains as suggested by the Reviewer. This caveat has been addressed on the main manuscript in page 7, lines 258-264: "It is notable that, the intensity signals from *low* and *high* order phases could be affected by interference between the two detection channels. Figure B in S5 Supporting Information shows the Laurdan dye emission spectra showing some level of overlap between the peaks for *high* and *low* order systems. Hence, it is possible that there is crosstalk between the emission signal of each phase. Nevertheless, the CLSM results align qualitatively with what was observable by GIWAXS and molecular dynamics simulations.”

Here we provide more details of the new Figure B in S5 Supporting Information:

We performed Laurdan spectral analysis in ambient environment for pure DPPC and DOPC lipids, which are representative of *high* and *low* order membranes at room temperature, respectively. Samples were excited with Xenon lamp excitation at λ_{ex} = 350 nm (bandpass: 20 nm) and the emission spectra was collected in n=3 measurements. DPPC at low temperature (25 °C) is assembled in a bilayer where hydrocarbon chains have a high degree of order yielding a Laurdan emission spectra with a maximum at ca. 443 nm. DOPC at 25 °C (above its transition temperature) comprises hydrocarbon tails with more conformational freedom yielding a peak with a maximum at ca 480 nm.

CL: Department of Materials Science and Engineering • MSEB, 1304 West Green St, Urbana, IL 61801 • E: cecilial@illinois.edu • T: 217-300-1955 • W: http://www.matse.illinois.edu/faculty/Leal.html
AZ: Department of Mechanical Science and Engineering • 214 Seitz Materials Research Lab, 104 South Goodwin Ave, Urbana, IL 61801 • E: arendv@illinois.edu • T: (217) 244-2912 • W: https://vanderzandelab.mechse.illinois.edu

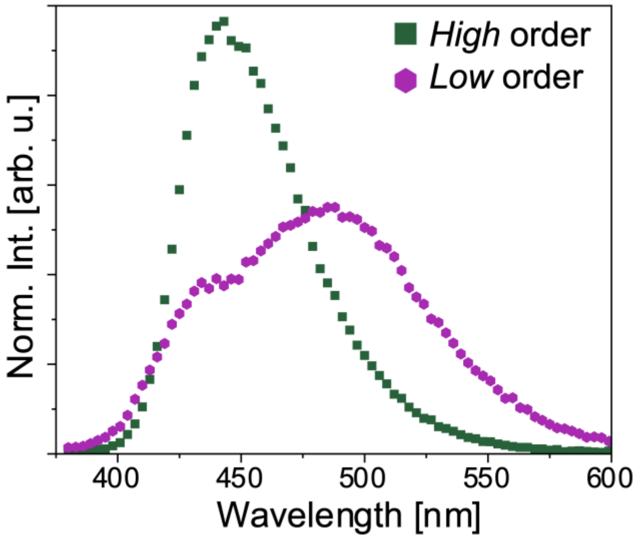

Figure B in S5 Supporting Information. Emission spectra of 1 mol% Laurdan in 50 mM DPPC and DOPC samples at 25 °C. Both samples were excited at 350 nm (20 nm bandpass) with Xenon lamp and corresponding spectra was collected using Synergy Neo 2 microplate reader (Biotek). DPPC has a *high* order phase Laurdan emission spectra with a peak at ca. 443 nm (colored in green). DOPC has a *low* order phase with a peak at ca. 480 nm (colored in magenta).

6) The conclusion of the paper is that nanoscale membrane undulations resulting from the compression are the main trigger for the change in membrane order. Since the delamination effects and buckling are dependent mainly on the membrane-substrate interaction, how is this interaction taken into account in the simulations on fig. 6? The authors may include a comment on the biological relevance of this interaction.

We appreciate the Reviewer for asking clarification on this matter. In this study, molecular simulations have been performed to probe the effect of compression on supported multilayered membranes at the nanoscale. In living systems, multilayered lipid structures are often supported onto polymeric flexible substrates such as actin filaments and the extracellular matrix, indicating the adaptive yet robust nature of these systems. Previous studies have explored the nanoscale effects of substrate on membrane properties [*new* references 69-71]. For supported lipid bilayers, a change in lipid morphology and dynamics has been reported only in the inner (closest to the substrate) lipid leaflet, whereas the outer leaflet properties were similar to a free-standing bilayer [71]. Hence, for the multilayered membranes considered in this study, the substrate effects are insignificant, and only the lipid-lipid interactions dictate the membrane properties.

Changes have been made in the main manuscript (page: 8, lines: 271-273, 288-296) to explain this.

CL: Department of Materials Science and Engineering • MSEB, 1304 West Green St, Urbana, IL 61801 • E: cecilial@illinois.edu • T: 217-300-1955 • W: http://www.matse.illinois.edu/faculty/Leal.html
AZ: Department of Mechanical Science and Engineering • 214 Seitz Materials Research Lab, 104 South Goodwin Ave, Urbana, IL 61801 • E: arendv@illinois.edu • T: (217) 244-2912 • W: https://vanderzandelab.mechse.illinois.edu
